# Comment on: “Treatment-resistant hiccups during general anesthesia possibly caused by remimazolam: a case report”—a reply

**DOI:** 10.1186/s40981-024-00738-9

**Published:** 2024-09-04

**Authors:** Yusuke Matsui, Tomonori Takazawa

**Affiliations:** 1 Department of Anesthesiology, National Hospital Organization Takasaki General Medical Center, 36 Takamatsu-Cho, Takasaki, 370-0829 Japan; 2 Department of Anesthesiology, Faculty of Medicine, University of Toyama, 2630 Sugitani, Toyama, 930-0194 Japan

To the Editor,

We thank Mizutani and Tsuchiya for their thoughtful commentary on our article [1]. They argued that hiccups in our case were induced because of the weak hypnotic effect and weak brainstem reflex inhibition under remimazolam, compared to propofol or inhaled anesthetics. We disagree with their argument for the following reasons.

Assuming that remimazolam induced the hiccups because it has a weak inhibitory effect on brainstem reflexes, in that case, additional doses of remimazolam should have reduced or eliminated the hiccups, but this did not happen. Furthermore, the hiccups persisted even after surgery but disappeared as the predicted blood level of remimazolam decreased [2]. These facts do not support their idea that hiccups were induced because remimazolam was less effective in suppressing brainstem reflexes, including hiccups.

They cited a paper stating that propofol is less effective at suppressing blink reflex activity, which is also a brainstem reflex, than sevoflurane, even at the same bispectral index (BIS) value [3]. Based on this paper, they suggested that remimazolam might also have a weaker inhibitory effect on the brainstem reflex of hiccups. However, the paper they cited was about sevoflurane and propofol and states nothing about remimazolam [3]. To justify their argument, they must show that remimazolam is a weak inhibitor of brainstem reflexes.

Indeed, the BIS value is a good reference for the depth of anesthesia but does not always help predict adverse reflexes during general anesthesia, including hiccups. In addition to BIS value, other vital signs, such as blood pressure and heart rate, should also be consulted to determine whether adequate sedation and analgesia are achieved during general anesthesia. In this case, since the patient’s vital signs did not suggest a lack of sedation and analgesia during anesthesia [2], we believe that the hiccups were not induced by inadequate sedation or analgesia.

In a study on sedation with remimazolam for endoscopic examination in elderly patients, hiccups occurred in eight of 64 patients (12.5%). This rate is much higher than the incidence of one out of 65 cases (1.5%) reported for propofol [4]. A recent randomized crossover trial showed a higher incidence of hiccups in patients with general anesthesia with remimazolam than with propofol (29% vs. 0%) [5]. Furthermore, hiccups caused by benzodiazepines, especially midazolam, which is very similar in action to remimazolam, have been reported several times in the past [6–8]. Hiccups after midazolam administration have been reported to disappear after flumazenil administration [9]. This evidence supports our hypothesis that like midazolam, remimazolam also has the potential to induce hiccups.

Our report is a single case report and does not demonstrate the mechanism by which remimazolam induces hiccups. Further research is needed to clarify the relationship between remimazolam and hiccups. In this case report, we emphasize that when hiccups occur during general anesthesia with remimazolam, anesthesiologists should consider using muscle relaxants or changing the anesthetic used.

## Data Availability

Data relevant to this case report are not available for public access because of patient privacy concerns but are available from the corresponding author on reasonable request.

## Abbreviation

BIS: Bispectral index
